# Innate Resistance and Susceptibility to Norovirus Infection

**DOI:** 10.1371/journal.ppat.1005385

**Published:** 2016-04-26

**Authors:** Johan Nordgren, Sumit Sharma, Anita Kambhampati, Ben Lopman, Lennart Svensson

**Affiliations:** 1 Division of Molecular Virology, IKE, Medical Faculty, Linköping University, Linköping, Sweden; 2 Division of Viral Diseases, Centers for Disease Control and Prevention, Atlanta, Georgia, United States of America; University of Kentucky, Lexington, UNITED STATES

The notion that certain individuals appear more or less susceptible to infections or to specific microbes is not new, but, until recently, it was assumed that clinical outcome of an infection was mainly owing to virulence factors of the microorganism. Relatively little attention has been given to host genetic factors involved in innate or adaptive immunity or expression of pathogen receptors. A remarkable example of susceptibility dependence is the strong Mendelian trait resistance to the most common noroviruses among individuals with a nonsense mutation in chromosome 19[1]. Norovirus is recognized as the leading cause of gastroenteritis worldwide, affecting children and adults alike [2]. Noroviruses are highly contagious and genetically diverse RNA viruses, but not all individuals are susceptible to infection to the same norovirus genotypes. Presence of histo-blood group antigens (HBGAs) on gut epithelial surfaces is essential for susceptibility to many norovirus genotypes. The synthesis of these HBGAs, specifically of the ABH and Lewis families, requires the use of several fucosyl and glycosyltransferases encoded by the *FUT2*, *FUT3*, and *ABH* genes. Polymorphisms in these genes vary considerably depending on ethnicity, with a homozygous nonsense mutation (individuals called non-secretors) in the *FUT2* gene occurring in approximately 5%–50% of different populations worldwide [3–5]. Secretor status also affects gut microbiota composition, including HBGA-expressing bacteria and bacteria inducing fucosylation in the gut. These could be intermediary factors that govern norovirus susceptibility [6–9].

## Does Secretor Status Mediate Resistance to Norovirus Infection?

Noroviruses infecting humans are highly diverse and comprise three genogroups and at least 33 genotypes, which are classified according to nucleotide identities in the major capsid protein gene. The earliest volunteer studies in the 1970s used the first isolated norovirus, the Norwalk virus (genogroup I, genotype 1, GI.1) [10]. These early studies hinted that not all individuals were inherently susceptible and, in the last decade, challenge and outbreak studies from several countries have confirmed a genetic component to norovirus susceptibility. Moreover, different norovirus genotypes are clearly associated with different epidemiological and susceptibility patterns. The globally dominant GII.4 viruses exhibit a strong secretor specificity in vivo [1]; furthermore, some data suggest that these viruses may possibly be more clinically severe [11,12]. Observational studies in countries such as the United States, Ecuador, Burkina Faso, Vietnam, China, and Nicaragua have consistently identified that non-secretors are highly resistant to GII.4 and that GII.4 variants emerging over time have similar secretor specificity in vivo, although in vitro studies have shown that some GII.4 variants can bind to sugars present in non-secretors [3,13–17].

Rarely, GII.4 noroviruses are found to infect non-secretors [14,16,18–20]. In the only GII.4 challenge study, non-secretors were significantly protected, but one became asymptomatically infected [19]. Likewise, both a GII.4 virus outbreak study in Spain and a prospective study in Burkina Faso identified GII.4 strains among non-secretors in one individual each [16,18]. Thus, although non-secretors are generally protected against GII.4 viruses, there is some evidence of both asymptomatic and symptomatic infections among non-secretors. The reasons for this are unresolved, but could be related to microbiota diversity, including activity of HBGA expressing bacteria [9].

Non-secretors are, however, susceptible to other norovirus strains. A challenge study using the Snow Mountain strain (genotype GII.2) showed no association between secretor status or ABH group antigens and susceptibility [21]. Two observational studies, one of a waterborne and another of a foodborne outbreak caused by genotype GI.3 strains in the Netherlands and Sweden, respectively [22,23], found no association between secretor status and susceptibility to symptomatic infection. Moreover, a birth cohort study in Ecuador with prospective follow-up found significantly higher rates of non-GII.4 norovirus infections in secretor-negative children [15], suggesting that the non-secretor geno/phenotype may increase susceptibility to certain norovirus genotypes.

Overall, several in vivo studies in various settings and countries have, with a few exceptions, consistently shown strong protection of non-secretors against GII.4 infections. However, non-secretors are susceptible to a wide array of other norovirus genotypes, the clinical and epidemiological significance of which is less well studied. No in vivo study has demonstrated specific norovirus genotypes that exclusively infect non-secretors, but rather genotypes that are secretor-independent. This further indicates that some norovirus genotypes use other primary ligands to infect.

## Do Populations with a Higher Proportion of Secretors Have Higher Rates of Norovirus Disease?

**Fig 1:**
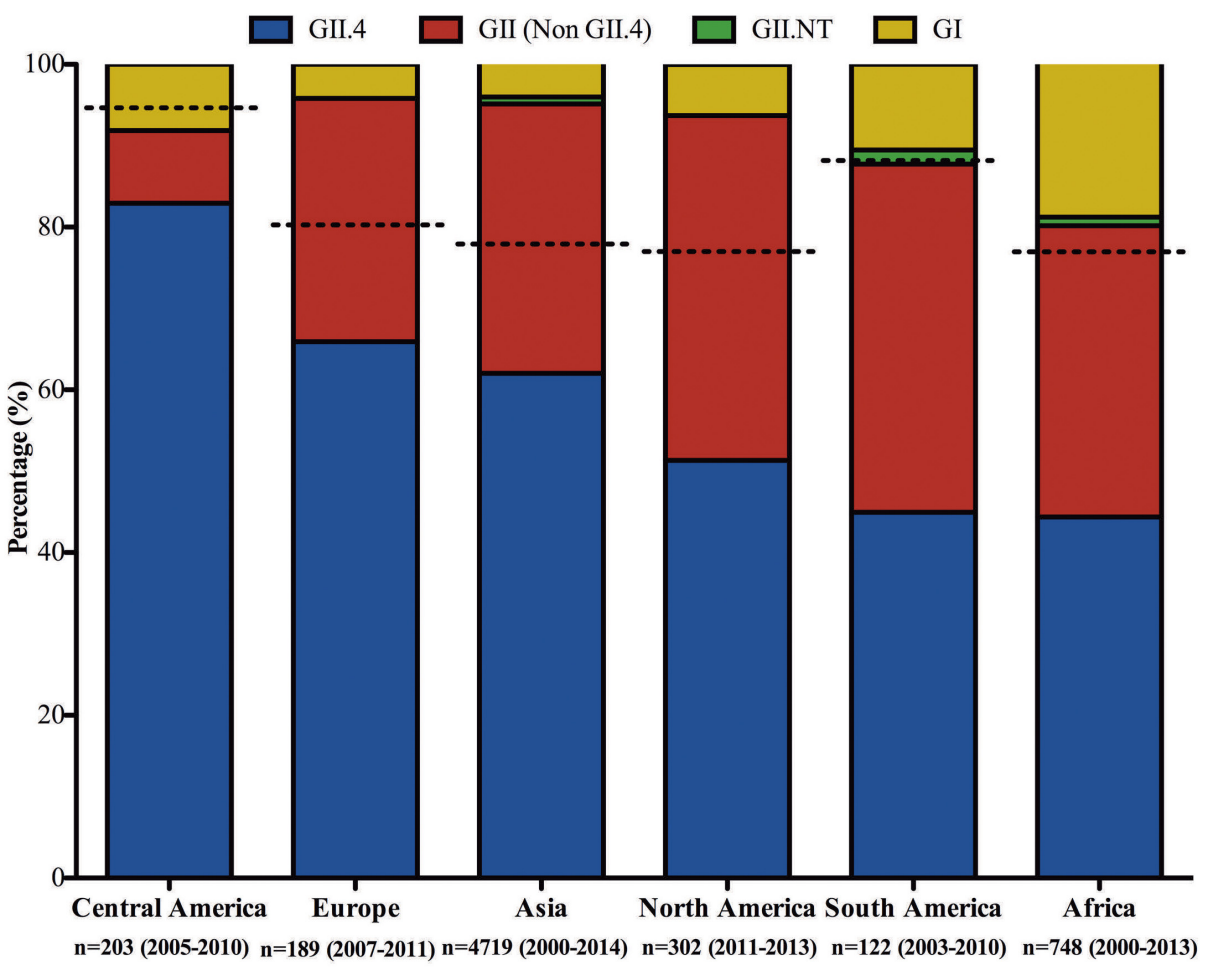
GII.4 detection rates worldwide among norovirus-positive children with sporadic acute gastroenteritis. Data were collected from 53 studies conducted in 31 different countries among children with sporadic acute gastroenteritis. Studies with samples collected after the year 2000 with sample collection done for a minimum of one year and based on genotyping of the capsid region are included. Due to lack of sufficient available data from Africa, studies conducted for less than one year have also been included for this region. The data shown are from a total of 6,283 genotyped samples from 28 different studies from Asia, 13 from Africa, five from South America, one from North America, and three each from Europe and Central America (S1 Table). The number (n) in the figure represents the number of genotyped samples, while the duration of sample collection for each region is mentioned within the parentheses. GII.NT represent non-typeables. Broken lines represent approximate percentages of secretors in the population.

Since distribution of the ABO(H), secretor, and Lewis genotypes is strongly dependent on ethnicity, we hypothesize that molecular epidemiology differs between regions because of host population genetics. According to this logic, populations with a higher proportion of secretors would be susceptible to both secretor-independent and dependent genotypes, including GII.4 virus. This could potentially translate into a higher norovirus infection rate than in populations with a lower proportion of secretors, especially since no norovirus genotypes have been found that selectively infect non-secretors. In European-descended, Asian, and some African populations, secretors constitute approximately 80% of the population (Fig 1). In contrast, secretor prevalence in Mesoamerican populations can be as high as 95% [3]. A high proportion of individuals genetically susceptible to GII.4 viruses might lead to higher infection rates with GII.4 among these populations. Indeed, Gll.4 noroviruses constitute 75% of infecting genotypes among children with diarrhea in community- and hospital-based studies from Nicaragua, Guatemala, and Mexico [12,24,25], a relatively higher proportion than the majority of studies from other continents (Fig 1). However, more studies are needed to address whether a higher frequency of GII.4 infections, or other secretor-dependent genotypes, also translates into higher disease burden, because the clinical relevance of many norovirus genotypes is not well established. The observation from Ecuador that norovirus gastroenteritis was similar, but genotype distribution differed between secretors and non-secretors, suggests that population-level risk may not scale in a simplistic way with secretor prevalence in all settings.

The dearth of population-based studies from a diverse range of settings, especially those with genetic testing of the host, precludes international comparisons of incidence and its relationship with population-level secretor genetics. In specific populations, for example, in the Philippines, Tanzania, and Saudi Arabia, the non-secretor phenotype can constitute upwards of 50% of the population [4]. Studies in these populations would provide important data to further understand the associations between secretor status, norovirus disease burden, and epidemiology at the population level.

## Are Noroviruses More Diverse in Human Populations with a Large Diversity of HBGA Polymorphisms?

The presence of different secretor and non-secretor human HBGAs is associated with increased susceptibility to several infectious diseases such as HIV, rhinovirus, *Haemophilus influenzae*, *Neisseria meningitidis*, and urinary tract infections [5], thus rendering a large selection of pressure interplay for the pathogens and perhaps resulting in genetic variation in the circulating pathogens as well as the human population. A reasonable hypothesis is that since human norovirus genotypes show differences in the spectrum of HBGAs to which they bind, it is possible that populations with a large diversity of HBGAs would sustain a greater variety of norovirus genotypes. Supporting this notion, genetic and phenotypic diversity of HBGAs has been found to be higher in sub-Saharan African populations compared to many other regions [16]. Accordingly, the norovirus genotype distribution in children is generally more diverse in Africa than other regions. For example, in the West African nation of Burkina Faso, all secretor, Lewis, and ABO phenotypes are present in relatively large proportions [16]. A prospective study there, spanning only ten months, detected 14 different norovirus genotypes in children with diarrhea. Other African studies have found similarly high norovirus diversity (Fig 1). This can be compared to the lower genotype diversity (approximately two to eight genotypes) generally found in similar studies performed in Europe and North America, where several HBGA phenotypes are present only in small proportions. More host genetic studies, especially from sub-Saharan Africa, are needed to address this.

In many Asian populations, the non-secretor phenotype is rare or non-existent. Instead, the weak-secretor genotype, which renders a low expression of secretor HBGAs, has approximately 15%–20% prevalence [26]. Weak-secretor children could be susceptible to several different norovirus genotypes, both secretor-dependent and secretor-independent, because they often express both secretor and non-secretor sugars simultaneously. GII.4 viruses have been found to symptomatically infect children and adults with weak-secretor geno/phenotypes in China [14,20], with weak-secretor children exhibiting partial protection [14].

## How Might the Presence of Non-secretor Individuals Affect Clinical Trial Design and Interpretation?

Norovirus vaccines are currently under development and have shown promise in early phase clinical trials that assessed protection against experimental virus challenge [27,28]. Vaccines that have entered human trials have been either monovalent (genotype GI.1) or bivalent (genotypes GI.1/GII.4). These vaccines have been shown to confer a degree of clinical efficacy against challenge with a genotype included in the vaccine. Other studies suggest that cross-reactive antibodies are produced after natural infection with certain noroviruses [29,30], but, to date, there are no clinical data on heterotypic protection from vaccination, although heterotypic blocking antibodies have been produced following intramuscular immunization with the bivalent vaccine [31].

Next, pivotal Phase III trials will be needed to measure protection against naturally acquired infection. In these trials, randomization should ensure that non-secretors are equally distributed amongst vaccine or placebo groups. However, measurement of vaccine efficacy (VE) may still be complicated if the subgroup of non-secretors differentially respond to vaccination and have a different risk of natural infection. To date, both GI.1 and GII.4 challenge trials have included only secretor-positive individuals [27,28], so we have no empirical data on whether non-secretors will gain protection through vaccination. In vaccine immunogenicity studies, despite having lower antibody levels at baseline, non-secretors appear to have similarly robust Pan-Ig responses to a single dose of parenteral vaccine containing both GI.1 and GII.4 virus-like particles (VLPs), compared to secretor individuals [32]. Perhaps non-secretors have been immunologically primed by infection with other norovirus strains, enabling a robust and broad response to the vaccine antigens. An optimistic interpretation of this observation of non-secretor humoral response is that they can gain protection by parenteral vaccination despite nearly complete resistance to natural infection with GI.1 or GII.4. However, parenteral administration of VLPs bypasses the mucosal epithelium; it may, therefore, produce a response unlike natural infection. To ensure protection for non-secretors, antibodies generated should be cross-reactive and provide mucosal protection against other (secretor-independent) noroviruses.

One possible finding of norovirus vaccine trials is that (a) non-secretors will respond similarly to vaccination (VE_sec_ = VE_non-sec_), and (b) vaccination will perform similarly against secretor-dependent noroviruses (e.g., GI.1 and GII.4) and secretor-independent noroviruses, so the distribution of non-secretors will not have a substantial influence on observed VE. On the other hand, if vaccination confers less protection against secretor-independent viruses (VE_sec_ > VE_non-sec_), the effect of the vaccine will be diluted in a population comprised of both secretors and non-secretors. In such a case, overall observed VE will be less than VE amongst secretors only and/or less than VE against secretor-dependent viruses only. This hypothetical issue goes beyond variation in type-specific efficacy, as has been observed for pneumococcal conjugate vaccine and other vaccines against vaccine types and non-vaccine types [33]. As there is also a human genetic component to norovirus susceptibility, there may be the potential for host-dependent variation in vaccine response, virus type-specific variation in vaccine protection, and an interaction between the two, because current vaccines are based on VLPs of secretor-dependent viruses.

On a practical level, we suggest that Phase III trials will be most informative if they test for interactions between vaccination and secretor status of enrolled subjects and consider infection with secretor-dependent norovirus genotypes as a secondary endpoint. For power calculations, it will be important to consider the prevalence of non-secretors in a trial population. If non-secretors have overall lower rates of norovirus illness, study power will be reduced with increased non-secretor prevalence in the trial population. If, however, incidence is the same, but infecting genotypes differ between secretors and non-secretors, as one cohort study suggests [15], overall VE may be a product of host infection history, host vaccine response, and virus type-specific protection, all of which may interact with host secretor status.

In summary, the interaction of noroviruses with secretor status provides a clear example of susceptibility to a pathogen being governed by genetics of the human host. Despite the unambiguous association, the interplay is complex, and implications for virus evolution, host disease risk, and interventions, such as vaccines, remain to be elucidated.

## Supporting Information

S1 Table Total number of samples genotyped and genotypes detected in each study that were used for generating Fig 1. (DOCX)
