# Supplementary material for: Innate Resistance and Susceptibility to Norovirus Infection
Source: PLoS Pathog. 2016 Apr 26;12(4):e1005385. doi: 10.1371/journal.ppat.1005385 (PMC4845991; doi:10.1371/journal.ppat.1005385)
Supplement: S1 Table — (DOCX) [file ppat.1005385.s001.docx]

Table S1: Total number of samples genotyped and genotypes detected in each study that were used for generating Figure 1.

| S. No. | Country | GII.4 | GII (Non GII.4) | GII.NT | GI | Total Samples genotyped | Reference Number |
| --- | --- | --- | --- | --- | --- | --- | --- |
| 1 | Bangladesh | 41 | 0 | 0 | 0 | 41 | (1) |
| 2 | China | 86 | 16 | 0 | 12 | 114 | (2) |
| 3 | China | 37 | 11 | 0 | 2 | 50 | (3) |
| 4 | China | 17 | 19 | 0 | 0 | 36 | (4) |
| 5 | China | 308 | 137 | 0 | 6 | 451 | (5) |
| 6 | China | 49 | 31 | 0 | 0 | 80 | (6) |
| 7 | China | 108 | 69 | 0 | 0 | 177 | (7) |
| 8 | China | 65 | 19 | 0 | 0 | 84 | (8) |
| 9 | China | 14 | 8 | 0 | 0 | 22 | (9) |
| 10 | Hong Kong | 155 | 37 | 1 | 2 | 195 | (10) |
| 11 | India | 12 | 7 | 0 | 2 | 21 | (11) |
| 12 | Japan | 20 | 37 | 0 | 1 | 58 | (12) |
| 13 | Japan | 19 | 32 | 0 | 0 | 51 | (13) |
| 14 | Japan | 56 | 48 | 0 | 0 | 104 | (14) |
| 15 | Japan | 664 | 266 | 0 | 8 | 938 | (15) |
| 16 | Japan | 117 | 69 | 0 | 6 | 192 | (16) |
| 17 | Nepal | 113 | 139 | 72 | 32 | 356 | (17) |
| 18 | South Korea | 82 | 20 | 0 | 12 | 114 | (18) |
| 19 | South Korea | 111 | 37 | 0 | 7 | 155 | (19) |
| 20 | South Korea | 74 | 42 | 0 |  | 116* | (20) |
| 21 | South Korea | 461 | 216 | 5 |  | 682* | (21) |
| 22 | South Korea | 195 | 83 | 7 | 17 | 302 | (22) |
| 23 | Taiwan | 41 | 8 | 0 | 7 | 56 | (23) |
| 24 | Thailand | 0 | 6 | 0 | 3 | 9 | (24) |
| 25 | Thailand | 22 | 13 | 0 | 0 | 35 | (25) |
| 26 | Thailand | 5 | 5 | 0 | 0 | 10 | (26) |
| 27 | Thailand | 59 | 30 | 0 | 1 | 90 | (27) |
| 28 | Vietnam | 168 | 12 | 0 | 0 | 180 | (28) |
| 29 | Burkina Faso | 10 | 18 | 2 | 7 | 37 | (29) |
| 30 | Egypt | 9 | 5 | 0 | 9 | 23 | (30) |
| 31 | Ghana | 6 | 4 | 0 | 3 | 13 | (31) |
| 32 | Jordan | 8 | 18 | 0 | 1 | 27 | (32) |
| 33 | Libya | 26 | 0 | 0 |  | 26* | (33) |
| 34 | Madagascar | 0 | 10 | 0 | 4 | 14 | (34) |
| 35 | Malawi | 40 | 12 | 0 | 16 | 68 | (35) |
| 36 | Morocco | 27 | 6 | 0 |  | 33* | (36) |
| 37 | South Africa | 147 | 86 | 0 | 27 | 260 | (37) |
| 38 | South Africa | 15 | 14 | 3 | 4 | 36 | (38) |
| 39 | Tunisia | 83 | 48 | 0 | 11 | 142 | (39) |
| 40 | Tunisia | 12 | 20 | 0 | 6 | 38 | (40) |
| 41 | Yemen | 8 | 11 | 0 | 12 | 31 | (41) |
| 42 | Brazil | 9 | 4 | 0 | 0 | 13 | (42) |
| 43 | Brazil | 20 | 11 | 3 | 0 | 34 | (43) |
| 44 | Brazil | 6 | 6 | 0 | 0 | 12 | (44) |
| 45 | Ecuador | 10 | 12 | 0 | 12 | 34 | (45) |
| 46 | Paraguay | 5 | 19 | 0 | 5 | 29 | (46) |
| 47 | Finland | 38 | 11 | 0 | 0 | 49 | (47) |
| 48 | Italy | 32 | 19 | 0 | 5 | 56 | (48) |

| S. No. | Country | GII.4 | GII (Non GII.4) | GII.NT | GI | Number genotyped all | Reference Number |
| --- | --- | --- | --- | --- | --- | --- | --- |
| 49 | Soviet Union | 53 | 28 | 0 | 3 | 84 | (49) |
| 50 | Guatemala | 62 | 10 | 0 | 12 | 84 | (50) |
| 51 | Mexico | 62 | 2 | 0 | 0 | 64 | (51) |
| 52 | Nicaragua | 45 | 7 | 0 |  | 55* | (52) |
| 53 | USA | 155 | 128 | 0 | 19 | 302 | (53) |

*: Percentages were adjusted proportionately as only a subset of GII and no GI were genotyped

References:

1. Dey SK, Nguyen TA, Phan TG, Nishio O, Salim AF, Rahman M, et al. Molecular and epidemiological trend of norovirus associated gastroenteritis in Dhaka City, Bangladesh. Journal of clinical virology : the official publication of the Pan American Society for Clinical Virology. 2007 Nov;40(3):218-23.

2. Jin M, Xie HP, Duan ZJ, Liu N, Zhang Q, Wu BS, et al. Emergence of the GII4/2006b variant and recombinant noroviruses in China. Journal of medical virology. 2008 Nov;80(11):1997-2004.

3. Jin Y, Cheng WX, Yang XM, Jin M, Zhang Q, Xu ZQ, et al. Viral agents associated with acute gastroenteritis in children hospitalized with diarrhea in Lanzhou, China. Journal of clinical virology : the official publication of the Pan American Society for Clinical Virology. 2009 Mar;44(3):238-41.

4. Zhang S, Chen TH, Wang J, Dong C, Pan J, Moe C, et al. Symptomatic and asymptomatic infections of rotavirus, norovirus, and adenovirus among hospitalized children in Xi'an, China. Journal of medical virology. 2011 Aug;83(8):1476-84.

5. Zeng M, Xu X, Zhu C, Chen J, Zhu Q, Lin S, et al. Clinical and molecular epidemiology of norovirus infection in childhood diarrhea in China. Journal of medical virology. 2012 Jan;84(1):145-51.

6. Sai L, Sun J, Shao L, Chen S, Liu H, Ma L. Epidemiology and clinical features of rotavirus and norovirus infection among children in Ji'nan, China. Virology journal. 2013;10:302.

7. Jia LP, Qian Y, Zhang Y, Deng L, Liu LY, Zhu RN, et al. Prevalence and genetic diversity of noroviruses in outpatient pediatric clinics in Beijing, China 2010-2012. Infection, genetics and evolution : journal of molecular epidemiology and evolutionary genetics in infectious diseases. 2014 Dec;28:71-7.

8. Tan D, Deng L, Wang M, Li X, Ma Y, Liu W. High prevalence and genetic diversity of noroviruses among children with sporadic acute gastroenteritis in Nanning City, China, 2010-2011. Journal of medical virology. 2015 Mar;87(3):498-503.

9. Ren Z, Kong Y, Wang J, Wang Q, Huang A, Xu H. Etiological study of enteric viruses and the genetic diversity of norovirus, sapovirus, adenovirus, and astrovirus in children with diarrhea in Chongqing, China. BMC infectious diseases. 2013;13:412.

10. Chan MC, Leung TF, Chung TW, Kwok AK, Nelson EA, Lee N, et al. Virus Genotype Distribution and Virus Burden in Children and Adults Hospitalized for Norovirus Gastroenteritis, 2012-2014, Hong Kong. Scientific reports. 2015;5:11507.

11. Nayak MK, Chatterjee D, Nataraju SM, Pativada M, Mitra U, Chatterjee MK, et al. A new variant of Norovirus GII.4/2007 and inter-genotype recombinant strains of NVGII causing acute watery diarrhoea among children in Kolkata, India. Journal of clinical virology : the official publication of the Pan American Society for Clinical Virology. 2009 Jul;45(3):223-9.

12. Phan TG, Kuroiwa T, Kaneshi K, Ueda Y, Nakaya S, Nishimura S, et al. Changing distribution of norovirus genotypes and genetic analysis of recombinant GIIb among infants and children with diarrhea in Japan. Journal of medical virology. 2006 Jul;78(7):971-8.

13. Phan TG, Kaneshi K, Ueda Y, Nakaya S, Nishimura S, Yamamoto A, et al. Genetic heterogeneity, evolution, and recombination in noroviruses. Journal of medical virology. 2007 Sep;79(9):1388-400.

14. Chan-It W, Thongprachum A, Khamrin P, Kobayashi M, Okitsu S, Mizuguchi M, et al. Emergence of a new norovirus GII.6 variant in Japan, 2008-2009. Journal of medical virology. 2012 Jul;84(7):1089-96.

15. Thongprachum A, Chan-it W, Khamrin P, Saparpakorn P, Okitsu S, Takanashi S, et al. Molecular epidemiology of norovirus associated with gastroenteritis and emergence of norovirus GII.4 variant 2012 in Japanese pediatric patients. Infection, genetics and evolution : journal of molecular epidemiology and evolutionary genetics in infectious diseases. 2014 Apr;23:65-73.

16. Yoneda M, Okayama A, Kitahori Y. Epidemiological characteristics of norovirus associated with sporadic gastroenteritis among children from the 2006/2007 to 2011/2012 season in Nara Prefecture, Japan. Intervirology. 2014;57(1):31-5.

17. Hoa-Tran TN, Nakagomi T, Sano D, Sherchand JB, Pandey BD, Cunliffe NA, et al. Molecular epidemiology of noroviruses detected in Nepalese children with acute diarrhea between 2005 and 2011: increase and predominance of minor genotype GII.13. Infection, genetics and evolution : journal of molecular epidemiology and evolutionary genetics in infectious diseases. 2015 Mar;30:27-36.

18. Yoon JS, Lee SG, Hong SK, Lee SA, Jheong WH, Oh SS, et al. Molecular epidemiology of norovirus infections in children with acute gastroenteritis in South Korea in November 2005 through November 2006. Journal of clinical microbiology. 2008 Apr;46(4):1474-7.

19. Chung JY, Han TH, Park SH, Kim SW, Hwang ES. Detection of GII-4/2006b variant and recombinant noroviruses in children with acute gastroenteritis, South Korea. Journal of medical virology. 2010 Jan;82(1):146-52.

20. Han TH, Kim CH, Chung JY, Park SH, Hwang ES. Emergence of norovirus GII-4/2008 variant and recombinant strains in Seoul, Korea. Archives of virology. 2011 Feb;156(2):323-9.

21. Cho HG, Lee SG, Kim JE, Yu KS, Lee DY, Park PH, et al. Molecular epidemiology of norovirus GII.4 variants in children under 5 years with sporadic acute gastroenteritis in South Korea during 2006-2013. Journal of clinical virology : the official publication of the Pan American Society for Clinical Virology. 2014 Nov;61(3):340-4.

22. Ham H, Oh S, Seung H, Jo S. Molecular characteristics of noroviruses genogroup I and genogroup II detected in patients with acute gastroenteritis. Annals of laboratory medicine. 2015 Mar;35(2):242-5.

23. Chen SY, Tsai CN, Lai MW, Chen CY, Lin KL, Lin TY, et al. Norovirus infection as a cause of diarrhea-associated benign infantile seizures. Clinical infectious diseases : an official publication of the Infectious Diseases Society of America. 2009 Apr 1;48(7):849-55.

24. Hansman GS, Katayama K, Maneekarn N, Peerakome S, Khamrin P, Tonusin S, et al. Genetic diversity of norovirus and sapovirus in hospitalized infants with sporadic cases of acute gastroenteritis in Chiang Mai, Thailand. Journal of clinical microbiology. 2004 Mar;42(3):1305-7.

25. Khamrin P, Maneekarn N, Peerakome S, Tonusin S, Malasao R, Mizuguchi M, et al. Genetic diversity of noroviruses and sapoviruses in children hospitalized with acute gastroenteritis in Chiang Mai, Thailand. Journal of medical virology. 2007 Dec;79(12):1921-6.

26. Khamrin P, Maneekarn N, Thongprachum A, Chaimongkol N, Okitsu S, Ushijima H. Emergence of new norovirus variants and genetic heterogeneity of noroviruses and sapoviruses in children admitted to hospital with diarrhea in Thailand. Journal of medical virology. 2010 Feb;82(2):289-96.

27. Chaimongkol N, Khamrin P, Malasao R, Thongprachum A, Kongsricharoern T, Ukarapol N, et al. Molecular characterization of norovirus variants and genetic diversity of noroviruses and sapoviruses in Thailand. Journal of medical virology. 2014 Jul;86(7):1210-8.

28. Trang NV, Luan le T, Kim-Anh le T, Hau VT, Nhung le TH, Phasuk P, et al. Detection and molecular characterization of noroviruses and sapoviruses in children admitted to hospital with acute gastroenteritis in Vietnam. Journal of medical virology. 2012 Feb;84(2):290-7.

29. Nordgren J, Nitiema LW, Ouermi D, Simpore J, Svensson L. Host genetic factors affect susceptibility to norovirus infections in Burkina Faso. PloS one. 2013;8(7):e69557.

30. Kamel AH, Ali MA, El-Nady HG, de Rougemont A, Pothier P, Belliot G. Predominance and circulation of enteric viruses in the region of Greater Cairo, Egypt. Journal of clinical microbiology. 2009 Apr;47(4):1037-45.

31. Armah GE, Gallimore CI, Binka FN, Asmah RH, Green J, Ugoji U, et al. Characterisation of norovirus strains in rural Ghanaian children with acute diarrhoea. Journal of medical virology. 2006 Nov;78(11):1480-5.

32. Kaplan NM, Kirby A, Abd-Eldayem SA, Dove W, Nakagomi T, Nakagomi O, et al. Detection and molecular characterisation of rotavirus and norovirus infections in Jordanian children with acute gastroenteritis. Archives of virology. 2011 Aug;156(8):1477-80.

33. Abugalia M, Cuevas L, Kirby A, Dove W, Nakagomi O, Nakagomi T, et al. Clinical features and molecular epidemiology of rotavirus and norovirus infections in Libyan children. Journal of medical virology. 2011 Oct;83(10):1849-56.

34. Papaventsis DC, Dove W, Cunliffe NA, Nakagomi O, Combe P, Grosjean P, et al. Norovirus infection in children with acute gastroenteritis, Madagascar, 2004-2005. Emerging infectious diseases. 2007 Jun;13(6):908-11.

35. Trainor E, Lopman B, Iturriza-Gomara M, Dove W, Ngwira B, Nakagomi O, et al. Detection and molecular characterisation of noroviruses in hospitalised children in Malawi, 1997-2007. Journal of medical virology. 2013 Jul;85(7):1299-306.

36. El Qazoui M, Oumzil H, Baassi L, El Omari N, Sadki K, Amzazi S, et al. Rotavirus and norovirus infections among acute gastroenteritis children in Morocco. BMC infectious diseases. 2014;14:300.

37. Mans J, Murray TY, Nadan S, Netshikweta R, Page NA, Taylor MB. Norovirus diversity in children with gastroenteritis in South Africa from 2009 to 2013: GII.4 variants and recombinant strains predominate. Epidemiology and infection. 2015 Sep 16:1-10.

38. Mans J, de Villiers JC, du Plessis NM, Avenant T, Taylor MB. Emerging norovirus GII.4 2008 variant detected in hospitalised paediatric patients in South Africa. Journal of clinical virology : the official publication of the Pan American Society for Clinical Virology. 2010 Dec;49(4):258-64.

39. Sdiri-Loulizi K, Ambert-Balay K, Gharbi-Khelifi H, Sakly N, Hassine M, Chouchane S, et al. Molecular epidemiology of norovirus gastroenteritis investigated using samples collected from children in Tunisia during a four-year period: detection of the norovirus variant GGII.4 Hunter as early as January 2003. Journal of clinical microbiology. 2009 Feb;47(2):421-9.

40. Hassine-Zaafrane M, Sdiri-Loulizi K, Kaplon J, Salem IB, Pothier P, Aouni M, et al. Prevalence and genetic diversity of norovirus infection in Tunisian children (2007-2010). Journal of medical virology. 2013 Jun;85(6):1100-10.

41. Kirby A, Al-Eryani A, Al-Sonboli N, Hafiz T, Beyer M, Al-Aghbari N, et al. Rotavirus and norovirus infections in children in Sana'a, Yemen. Tropical medicine & international health : TM & IH. 2011 Jun;16(6):680-4.

42. Barreira DM, Ferreira MS, Fumian TM, Checon R, de Sadovsky AD, Leite JP, et al. Viral load and genotypes of noroviruses in symptomatic and asymptomatic children in Southeastern Brazil. Journal of clinical virology : the official publication of the Pan American Society for Clinical Virology. 2010 Jan;47(1):60-4.

43. Sa AC, Gomez MM, Lima IF, Quetz JS, Havt A, Oria RB, et al. Group a rotavirus and norovirus genotypes circulating in the northeastern Brazil in the post-monovalent vaccination era. Journal of medical virology. 2015 Sep;87(9):1480-90.

44. Aragao GC, Mascarenhas JD, Kaiano JH, de Lucena MS, Siqueira JA, Fumian TM, et al. Norovirus diversity in diarrheic children from an African-descendant settlement in Belem, Northern Brazil. PloS one. 2013;8(2):e56608.

45. Lopman BA, Trivedi T, Vicuna Y, Costantini V, Collins N, Gregoricus N, et al. Norovirus Infection and Disease in an Ecuadorian Birth Cohort: Association of Certain Norovirus Genotypes With Host FUT2 Secretor Status. The Journal of infectious diseases. 2015 Jun 1;211(11):1813-21.

46. Galeano ME, Martinez M, Amarilla AA, Russomando G, Miagostovich MP, Parra GI, et al. Molecular epidemiology of norovirus strains in Paraguayan children during 2004-2005: description of a possible new GII.4 cluster. Journal of clinical virology : the official publication of the Pan American Society for Clinical Virology. 2013 Oct;58(2):378-84.

47. Puustinen L, Blazevic V, Salminen M, Hamalainen M, Rasanen S, Vesikari T. Noroviruses as a major cause of acute gastroenteritis in children in Finland, 2009-2010. Scandinavian journal of infectious diseases. 2011 Oct;43(10):804-8.

48. Medici MC, Tummolo F, Martella V, Chezzi C, Arcangeletti MC, De Conto F, et al. Epidemiological and molecular features of norovirus infections in Italian children affected with acute gastroenteritis. Epidemiology and infection. 2014 Nov;142(11):2326-35.

49. Chhabra P, Samoilovich E, Yermalovich M, Chernyshova L, Gheorghita S, Cojocaru R, et al. Viral gastroenteritis in rotavirus negative hospitalized children <5 years of age from the independent states of the former Soviet Union. Infection, genetics and evolution : journal of molecular epidemiology and evolutionary genetics in infectious diseases. 2014 Dec;28:283-8.

50. Estevez A, Arvelo W, Hall AJ, Lopez MR, Lopez B, Reyes L, et al. Prevalence and genetic diversity of norovirus among patients with acute diarrhea in Guatemala. Journal of medical virology. 2013 Jul;85(7):1293-8.

51. Gomez-Santiago F, Ribas-Aparicio RM, Garcia-Lozano H. Molecular characterization of human calicivirus associated with acute diarrheal disease in Mexican children. Virology journal. 2012;9:54.

52. Bucardo F, Reyes Y, Svensson L, Nordgren J. Predominance of norovirus and sapovirus in Nicaragua after implementation of universal rotavirus vaccination. PloS one. 2014;9(5):e98201.

53. Currier RL, Payne DC, Staat MA, Selvarangan R, Shirley SH, Halasa N, et al. Innate Susceptibility to Norovirus Infections Influenced by FUT2 Genotype in a United States Pediatric Population. Clinical infectious diseases : an official publication of the Infectious Diseases Society of America. 2015 Jun 1;60(11):1631-8.
